# Patterns and Grading of Gastrointestinal Graft-Versus-Host Disease: A Clinicopathologic Correlation Study

**DOI:** 10.5152/tjg.2023.22012

**Published:** 2023-05-01

**Authors:** Sonay Kuş Öztürk, Ayça Kırmızı, Nermin Aras, Cevriye Cansız Ersöz, Meltem Kurt Yüksel, Elif Ünal İnce, Berna Savaş, Mehmet Ertem, Aydan Kansu, Hülya Çetinkaya, Arzu Ensari

**Affiliations:** 1 Department of Pathology, Ankara University Faculty of Medicine, Sıhhıye, Ankara, Turkey; 2 Department of Hematology, Ankara University Faculty of Medicine, Sıhhıye, Ankara, Turkey; 3 Department of Pediatric Hematology, Ankara University Faculty of Medicine, Sıhhıye, Ankara, Turkey; 4 Department of Pediatric Gastroenterology, Ankara University Faculty of Medicine, Sıhhıye, Ankara, Turkey; 5 Department of Gastroenterology, Ankara University Faculty of Medicine, Sıhhıye, Ankara, Turkey

**Keywords:** Acute graft-versus-host disease, clinical grading, endoscopic grading, gastrointestinal tract, pathologic grading

## Abstract

**Background:**

The present study investigated gastrointestinal involvement patterns of acute graft-versus-host disease and assessed the correlation of pathologic severity with clinical grading.

**Methods:**

Pathology reports of gastrointestinal (GI) endoscopic biopsies taken from 164 post-hematopoietic stem cell transplant patients with at least 1 endoscopic gastrointestinal biopsy diagnosed as “consistent with acute graft-versus-host disease” between 2005 and 2019 were retrieved from the automated hospital database. Endoscopic, pathologic and clinical gradings were performed using Freiburg criteria, Lerner and modified Seattle-Glucksberg grading systems, respectively.

**Results:**

The majority of the patients (n = 140, 85.4%) were investigated with more than one biopsy from various gastrointestinal sites with a total of 479 biopsies: 44 (9.2%) esophagus, 90 (18.8%) stomach, 91 (19.0%) duodenum, 20 (4.2%) terminal ileum, 32 (6.7%) right colon, 87 (18.2%) left colon and, 115 (23.9%) rectum. Overall, lower gastrointestinal (n = 118/126, 93.6%) and upper gastrointestinal (n = 91/97, 93.8%) involvements were similar (*P* = .3). While the most severely affected site was duodenum (*P* = .021) in upper gastrointestinal, pathologic grades were similar in lower gastrointestinal sites, though more severe than upper gastrointestinal (*P* = .003). Pathologic grading had a low positive correlation with both clinical (*r* = 0.308, *P* = .001) and endoscopic grading (coefficient: 0.261, *P* = .003).

**Conclusion:**

Considering the similar graft-versus-host disease frequency of upper and lower gastrointestinal tract, distal colon evaluation with rectosigmoidoscopy seems to be a practical approach in patients with suspected gastrointestinal graft-versus-host disease. As it was positively correlated with both endoscopic and clinical grade, pathologic grading should be performed in these patients to assess gastrointestinal involvement patterns.

**Main Points**

- Although graft-versus-host disease has a tendency to lower gastrointestinal tract, duodenal graft-versus-host disease should not be ignored with a similar graft-versus-host disease involvement as lower gastrointestinal tract.
- The similar involvement rate of graft-versus-host disease throughout lower gastrointestinal makes proctosigmoidoscopy the most practical initial approach in graft-versus-host disease diagnosis.
- Nonspecific microscopic findings of graft-versus-host disease oblige an integrated approach consisting of clinical, endoscopic, and pathologic evaluation.

## Introduction

Graft-versus-host disease (GVHD) is a serious condition which may occur following allogeneic bone marrow or peripheral hematopoietic stem cell transplantation (HSCT). Underlying pathogenetic mechanism involves the allo-immunoreaction caused by engrafted donor immune cells resulting in epithelial cell apoptosis, inflammation, and tissue injury.^1^

Acute GVHD usually occurs within the first 100 days after transplantation, whereas chronic GVHD occurs later. Due to the presence of late-onset acute GVHD, the discrimination of acute and chronic GVHD should not solely depend on the time of GVHD emergence as clinical manifestations are more important indicators of distinction of acute from chronic GVHD.^2^ In acute GVHD, liver, skin, gastrointestinal tract (GIT), bone marrow, thymus, and lung can be affected. Gastrointestinal tract is the second most frequently affected part of the body in acute GVHD^3^ presenting mainly with nonspecific symptoms like diarrhea, nausea, and vomiting which can also be seen in infectious gastroenteritis (i.e., Cytomegalovirus) or drug toxicity. Thereby, histopathological examination of GI biopsy is crucial for accurate diagnosis.^4-6^

Acute GVHD is an emergency diagnosis for pathologists but assessment of GVHD in GIT is not always easy. Considering that apoptosis, the main histopathologic feature of GVHD, could be seen in various situations, diagnosis may become a nightmare, particularly, when a superimposed GI infection with CMV is present. Therefore, the pathologist should be cautious when making a diagnosis of acute GVHD unless there are florid abnormalities in the biopsy together with sufficient clinical and endoscopic information.^4^

Histopathological evaluation of GVHD requires grading of tissue changes reflecting the severity of disease. Grading of GI GVHD with increasing severity consists of apoptosis, crypt damage, crypt loss, and mucosal shedding initially defined by Lerner et al.^7^ On the basis of this grading system, it has been reported that lower GI, mainly rectum and left colon are the most severely and frequently affected sites by GVHD^8-13^ while duodenum is the most commonly affected part in the upper GIT.^13^ The 2014 Consensus Conference on the histopathology of GVHD revised histopathologic criteria of acute and chronic GVHD with no recommendation for a grading scheme or for a particular biopsy site in the GI tract. It was suggested, however, that, if an institution prefers to use a grading system, the site with the greatest damage and grade should be reported.^4^ Clinical grading of acute GVHD, on the other hand, is performed using the updated version of the Glucksberg^14^ and the International Bone Marrow Transplant Registry grading systems proposed by Przepiorka et al^15^ which is based on affected organ system including GIT, skin, and liver together with the severity of the damage.^14,15^ For the endoscopic grading of intestinal acute GVHD Freiburg Criteria based on the severity of mucosal damage are widely employed.^16,17^ Clinical symptoms and endoscopic findings are very helpful in the work-up of GI GVHD when histopathologic examination supports the diagnosis, however, clinicopathologic correlation is not always present. A limited number of studies looked at the correlation of clinical and pathologic grading systems and found no or low correlation between clinical and pathological grades of GI GVHD.^5,12^ The correlation of endoscopic and pathologic grades also remains controversial as some studies support their concordance^16-18^ while others reveal a discordance.^19,20^

In the present study, we aimed to investigate gastrointestinal (GI) involvement patterns of acute GVHD in different sites of the GIT and to assess their value in terms of clinicopathologic correlation.

## MATERIALS AND METHODS

### Patients

Pathology reports of GI endoscopic biopsies taken from post-hematopoietic stem cell transplant (HSCT) patients between 2005 and 2019 were retrieved from the automated hospital database of the Department of Pathology, Ankara University Faculty of Medicine. A total of 479 GI biopsies obtained from 164 patients with at least one GI biopsy diagnosed as “consistent with acute GVHD” by 2 GI pathologists (AE, BS) were included in the study. Patients with repeated biopsies were excluded. The majority of the patients (n = 140, 85.4%) were investigated with more than 1 biopsy obtained from various GI sites while the remaining (n = 24, 14.6%) had only 1 biopsy taken mostly from the left colon (n = 20). Clinical information comprising age, sex, underlying disease, graft types, conditioning regimens, stem cell source, donor type, tissue match, presence of GVHD in other organ systems, endoscopic findings, GI symptoms, and time between transplantation and biopsy were obtained from the patients’ electronic medical records and manual registries of endoscopy units. The study complies with the Ethical Standards and found convenient by the institutional review board of Ankara University Faculty of Medicine (Ref. no: 2019/1).

### Histopathologic Evaluation and Grading

**Figure 1.**
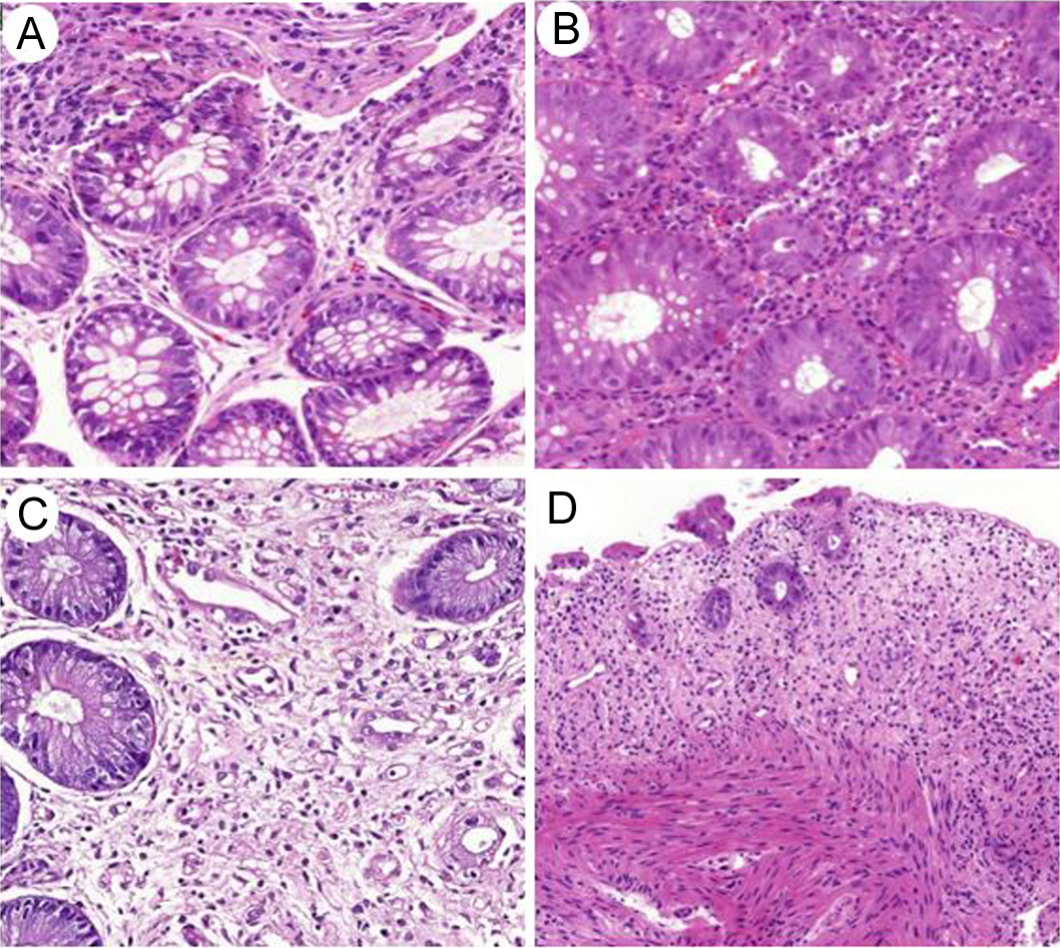
Histologic grading of GVHD. (A) Grade 1, apoptosis in crypt epithelium (H&E, ×200); (B) grade 2, isolated crypt destruction (H&E, ×200); (C) grade 3, contiguous crypt destruction (H&E, ×200); (D) grade 4, diffuse mucosal denudation/ulceration (H&E, ×40). GVHD, graft versus host disease; H&E, hematoxylin and eosin.

At the time of diagnosis, GI biopsies were evaluated by experienced GI pathologists (AE, BS) who were critical regarding differential diagnosis of GVHD including chemoradiation toxicity, medication side effects, and concurrent infections which were all excluded with careful histopathological analysis. Grading of acute GVHD was made using the worldwide accepted system of Lerner et al^7^ and NIH Consensus Criteria.^21^ The grading system classifies the severity of the disease into 4 groups (Figure 1) including grade 1: apoptosis of gland/crypt epithelium; grade 2: apoptosis of epithelial cells with isolated gland/crypt destruction; grade 3: apoptosis of epithelial cells with extensive gland/crypt destruction leading to crypt loss; grade 4: total mucosal denudation/ulceration. Apoptosis was more prominent in the regenerative compartment of the gland/crypt: in the gastric body, apoptotic cells were found primarily in the neck area of the glands and in the deeper glands in antrum. In the duodenum and other small bowel sites, apoptotic bodies were more prominent in the neck and deep crypts than in the villous epithelium while they were confined to deep colonic crypts in the large intestines. CMV infection was suspected in 62 cases which were investigated with immunohistochemistry using anti-CMV antibody (clone DDG9/CCH2, Cell Marque, Rocklin, Calif) and streptavidin Biotin-peroxidase technique on Benchmark XT automated stainer (Ventana, Tucson, AZ).

### Clinical and Endoscopic Grading

The clinical grading of acute GVHD was performed using the modified Seattle––Glucksberg grading system^14^ which is based on staging the most severely affected organs consisting of skin, GIT, and liver. While the extent of maculopapular rash and serum bilirubin levels were taken into consideration to determine the clinical stage for skin and liver, respectively, stool volume or presence of severe abdominal pain, ileus, grossly bloody stool were assessed to determine the clinical GI stage. According to the stages given to these 3 organs, namely, skin, liver, and GIT, an overall clinical GVHD grade of the patient was obtained (Table 1).

Endoscopic grading was done according to Freiburg criteria, a 4-tier classification system: Grade 1: no clear-cut criteria; grade 2: spotted erythema; grade 3: aphthous lesions; and grade 4: confluent defects, ulcers, denudation of the mucosa.^16,17^

### Statistical Analysis

Analysis of nonparametric variables were done with either Chi-square test or Mann–Whitney U test. Spearman’s rank correlation coefficient test was used to detect the correlations between clinical, pathologic, and endoscopic grades. For statistical purposes, comparisons were repeated by grouping cases as low (grades 1 and 2) and high (grades 3 and 4) grades. Statistical Package Social Sciences version 25.0 (IBM Corp., Armonk, NY, USA) was used for all analysis and *P* <.05 was considered statistically significant.

## Results

### Clinical Features

Our series consisted of 164 patients comprising 133 (81.1%) adults and 31 (18.9%) children/adolescents (age <18 years) with a median age of 38 years (age range: 1-68 years) and a male predominance of 7 to 5 (n = 97, 59.1% males; n = 67, 40.9% females). More than half of the patients had bone marrow transplantation for acute leukemia (n = 90, 54.9%), followed by myelodysplastic syndrome (n = 18, 11.0%), chronic leukemia (n = 15, 9.1%), and multiple myeloma (n = 3, 1.8%). Other diseases consisted of Hodgkin’s or non-Hodgkin’s lymphomas, idiopathic myelofibrosis, thalassemia major, aplastic anemia, autoimmune hemolytic anemia, sickle cell anemia, hemophagocytic lymphohistiocytosis, severe common immunodeficiency, and cirrhosis. The majority of all grafts were allogeneic (n = 153, 93.3%), while only 6 were autologous and 2 were liver transplants. A significant proportion of the patients (n = 112, 68.3%) had myeloablative conditioning regimens and peripheral blood was used as a stem cell source in 72.6% (n = 119). Symptomatology was known in almost all of the patients (n = 163, 99.4%) and diarrhoea was the leading symptom in 44.8% (n = 73) of the patients followed by both upper and lower GI symptoms (28.8%; n = 47), and 26.4% (n = 43) upper GI symptoms. Endoscopic findings were extracted from the endoscopy reports in 128 out of 164 patients. According to the reports: 50 (39.0%) patients had normal mucosa (Grade 1), 27 (21.0%) showed erythema (Grade 2), 32 (25.0%) had aphthous lesions (Grade 3), and 19 (15.0%) presented with ulceration (Grade 4). Clinical features of the patients are presented in Table 2.

### Histopathologic Findings

A total of 479 biopsies were obtained from 164 patients included in the study. The distribution of biopsies taken from different GI sites was as follows: 44 (9.2%) esophagus, 90 (18.8%) stomach, 91 (19.0%) duodenum, 20 (4.2%) terminal ileum, 32 (6.7%) right colon, 87 (18.2%) left colon and, 115 (23.9%) rectum. Since 78 cases had simultaneous biopsies from left colon and rectum, they were grouped together for statistical purposes making a total of 124 biopsies.

Majority of the patients who were investigated with multiple biopsies (n = 140, 85.4%) had biopsy-proven GI GVHD in more than one site (n = 115/140, 82.1%) and one-half of these patients (n = 72/140, 51.4%) had biopsy-proven GI GVHD in all sampled regions. In cases with simultaneous upper and lower GI biopsies, GVHD involvement of the lower GI, upper GI, and, both upper and lower GI was found in 13.5%, 10.1%, and 76.2% of the cases, respectively.

**Figure 2.**
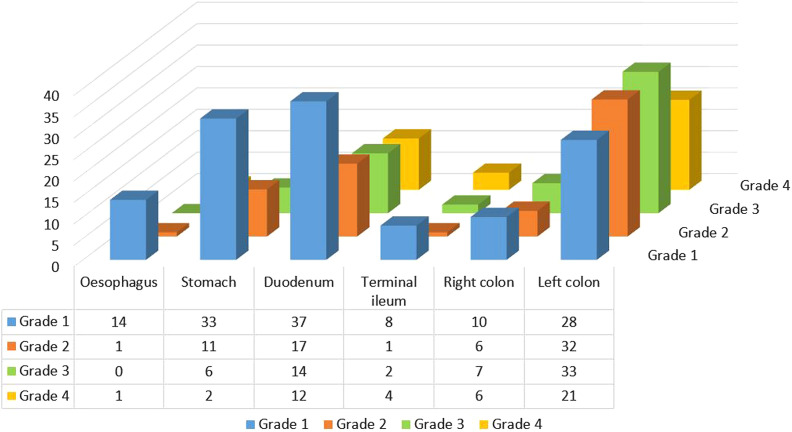
Pathologic GVHD grades along the GIT. GVHD, graft versus host disease; GIT, gastrointestinal tract.

Due to the high frequency of duodenal GVHD (n = 80/91, 87.9%), overall LGI (n = 118/126, 93.6%) and UGI (n = 91/97, 93.8%) involvements were similar (*P* = .3). Duodenum (n = 80/91, 87.9%) was the most frequently (*P* < .0001) involved site in upper GI followed by stomach (n = 52/90, 57.8%), and esophagus (n = 16/44, 36.4%) while in the lower GIT both right (n = 29/32, 90.6%) and left colon including rectum (n = 114/124, 91.9%) were equally affected with higher frequency, followed by terminal ileum (n = 15/20, 75%). The severity of GVHD in the duodenum and lower GI was higher than GVHD in the stomach (*P* = .021; *P* = .003, respectively) and esophagus (*P* = .005; *P* = .001, respectively) (Figure 2).

Correlation analysis of pathologic GVHD grades in different GI sites revealed that only esophagus and stomach had a weak positive correlation (coefficient: 0.334, *P* = .029) in upper GI while no such correlation was found for the lower GI sites. In lower GI, terminal ileum had a moderate positive correlation with right (coefficient: 0.658, *P* = .003) and left colon (coefficient: 0.540, *P* = .021) similar to the correlation between right and left colon (coefficient: 0.649, *P* < .000).

### Clinicopathologic Findings

In order to evaluate the relation between pathologic GVHD grades and clinical features, the highest GVHD grade was accepted as the pathologic grade for each patient. Accordingly, the distribution of pathologic GVHD grades was as follows: 46 (28.0%) grade 1, 42 (25.6%) grade 2, 44 (26.8%) grade 3, and 32 (19.5%) grade 4. Statistical analysis disclosed that detection rate of GVHD in more than one localization increased in parallel with pathologic GVHD grades (*P* < .001). The frequencies of UGI and LGI involvements were similar in patients with UGI symptoms, LGI symptoms, and both upper and lower GI symptoms. Though not significant, with the rise in pathologic GVHD grade, the frequency of sole upper GI symptoms (nausea and vomiting) decreased, whereas the coexistence of upper and lower GI symptoms (nausea, vomiting, and diarrhoea) increased (*P* = .077). Other clinical features like age, sex, donor type, underlying disease, conditioning regimens, and tissue match did not have any significant relationship with pathologic GVHD grade. When same comparisons were made by classifying the cases as low grade (grades 1 and 2) and high grade (grades 3 and 4) GVHD, similar results were obtained.

The clinical GI stages obtained from 113 patients were distributed as follows: 22 (19.5%) stage 0, 19 (16.8%) stage 1, 39 (34.5%) stage 2, 23 (20.4%) stage 3, and 10 (8.8%) stage 4. A low positive correlation was found between the clinical GI stage and highest pathologic GVHD grade (coefficient: 0.308, *P* = .001). Correlation analysis for pathologic and endoscopic grades revealed a weak positive correlation (coefficient: 0.261, *P* = .003). However, no such correlation was found between endoscopic grades and clinical GI stages (coefficient: 0.100, *P* = .3).

Extra-GI GVHD was suspected in the clinical follow-up of some patients and extra-GI biopsies were taken due to their changing symptomatology.

Biopsy-proven extra-GI GVHD comprising skin (n = 65, 39.6%), liver (n = 21, 12.8%), oral mucosa (n = 14, 8.5%), eye (n = 15, 9.1%), and lung GVHDs (n = 2, 1.2%) was found in 71.3% (n = 117) of the patients. CMV immunohistochemical staining in pathologically or clinically suspected 62 patients was entirely negative.

## Discussion

The results of the present study revealed that GI GVHD predominantly and more severely involved the lower GIT and duodenum with increased severity compared to other upper GI sites. Furthermore, all lower GI sites were invariably involved while uneven distribution was observed in the upper GIT with more common duodenal involvement compared to esophagus and stomach. Studies published so far have mainly highlighted distal colon (rectum or sigmoid colon) involvement as the most common pattern of GI GVHD,^8,9,11,12,22^ whereas few have reported upper GI involvement being more frequent^13^ or equal to lower GI.^23,24^ It was also shown that proximal and distal colon were equally affected by GVHD prompting some researchers to perform proctosigmoidoscopy as the first line investigation.^9,10,13,23-26^ Based on predominant and diffuse lower GIT involvement by GVHD, our results confirm prior studies and suggest that distal colon investigation would be an effective approach to diagnose GI GVHD in most cases lowering the risks associated with a detailed endoscopic examination. This also holds true for cases with sole upper GI symptoms, as regardless of the symptoms, a similar GVHD involvement in UGI and LGI tract was observed. On the other hand, some investigators suggested that GI GVHD may present with a pan-intestinal or “patchy-diffuse” pattern disease^3,13,23,24,27-30^ which was also confirmed in the present study where the majority of patients with simultaneous upper and lower GI biopsies had GVHD in both. Therefore, a full GI endoscopy should be considered, particularly in patients diagnosed as “not compatible with GVHD” with single-site biopsies in order not to miss such cases with “patchy” disease. Considering the high involvement rate compared to esophagus and stomach, duodenum should also be carefully investigated especially in patients with a lower GI biopsy.

There are limited number of studies comparing the severity of GVHD in different GI sites (see Table 3). According to Ma et al.^9^ stomach was the least whereas rectum was the most severely affected location in GIT. Pathologic grades were similar at different sites in upper GI, in contrast to lower GI where a significant increase in grades from proximal to distal colon was observed.^9^ Conversely, Nomura et al^25^ comparing only lower GI locations found that severity increased from left to right colon and terminal ileum. Our results partly agree with the former study as the least severely affected sites were the stomach and esophagus, but the severity was similar within lower GIT locations. While a poor positive correlation was found between esophagus and gastric GVHD grades, duodenum presented similar GVHD grades to the colon. Thus, confirming that, duodenum, as the most commonly and severely affected site of upper GI, is the main biopsy site that should be carefully investigated, particularly when only upper GI endoscopy is performed. In lower GI, however, all regions had a moderate positive correlation with each other in terms of severity.

Conflicting results were found in studies comparing pathologic and clinical grades as some researchers found no or low concordance^5,12^ and proposed that pathologic grading of GVDH should not be considered as a compulsory practice, whereas others observed a high correlation between pathologic and clinical grades.^31-33^ Contradictory results were also shown in studies comparing pathologic and endoscopic grades.^16-20^ According to our results, pathologic grades had low concordance with clinical and endoscopic grades. Behind this low concordance, there would be several reasons including possible patchy distribution of the disease in GIT, lack of standardized biopsy protocols, or the early endoscopic biopsy protocol in the clinical work-up of GVHD in our institute before the clinical picture is complete. On the other hand, these results highlight the significance of pathological examination in GVHD diagnosis in GIT.

Retrospective nature and the lack of standardized endoscopic biopsy protocol constitute the major limitations of our study. Had the biopsies contained every GI site in every patient, the comparisons of these sites in terms of prevalence and severity of pathology would be optimal. On the other hand, however, the thorough clinical and histopathological evaluation of such a large cohort from a single institution make the results of the study noteworthy.

In conclusion, the results of the present study suggest that distal colon evaluation with rectosigmoidoscopy together with upper GI endoscopy, duodenoscopy in particular, would be the initial and probably sufficient approach in patients with suspected GI GVHD since duodenum and lower GIT are similarly affected by GVHD in terms of frequency and severity. However, due to possible “patchy-diffuse” nature of the disease, GVHD diagnosis cannot be ruled out with negative left colon and duodenum biopsies before performing a full colonoscopy and esophagogastroduodenoscopy with multiple site biopsies. Finally, as GI GVHD presents with nonspecific features, a clinical, endoscopic, and pathologic evaluation should be made by an experienced medical team comprising hemato-oncologists, gastroenterologists, and GI pathologists for an accurate diagnosis.

## Figures and Tables

**Table 1.** Clinical Stage of Acute GVHD^14^

Stage	Target Organ			
	Skin (Active Erythema Only)	Liver (Serum Total Bilirubin)	Upper Gastrointestinal	Lower Gastrointestinal (Stool Output)
0	No active (erythematous) rash	<2 mg/dL (<34.21 µmol/L)	No or intermittent nausea, vomiting, or anorexia	Adult: <500 mL per day Child: <10 mL/kg per day
1	Maculopapular rash, <25% BSA	2-3 mg/dL (34.21-51.31 µmol/L)	Persistent nausea, vomiting, or anorexia	Adult: 500-999 mL per day Child: 10-19.9 mL/kg per day
2	Maculopapular rash, 25%-50% BSA	3.1-6 mg/dL (53.02-102.62 µmol/L)	–	Adult: 1.000-1.500 mL/day Child: 20-30 mL/kg/day
3	Maculopapular rash, >50% BSA	6.1-15 mg/dL (104.33-256.56 µmol/L)	–	Adult: >1.500 mL per day Child: >30 mL/kg per day
4	Generalized erythroderma (>50% BSA), plus bullous formation and desquamation (>5% BSA)	>15 mg/dL (>256.56 µmol/L)	–	Severe abdominal pain with or without ileus or grossly bloody stool (regardless of volume)

BSA, body surface area.

**Table 2.** Clinical Features of the Patients

Patient characteristics	n (%)
Age (years), median (range)	38 (1-68)
Sex	
Female	67 (40.9)
Male	97 (59.1)
Underlying disease	
Acute myeloid leukemia	56 (34.1)
Acute lymphoid leukemia	34 (20.7)
Chronic leukemia	15 (9.1)
Myelodysplastic syndrome	18 (11.0)
Multiple myeloma	3 (1.8)
Other	35 (21.3)
Unknown	3 (1.8)
Graft types	
Allogeneic	153 (93.3)
Autologous	6 (3.7)
Solid organ	2 (1.2)
Unknown	3 (1.8)
Conditioning regimens	
Myeloablative	112 (68.3)
Non-myeloablative	15 (9.1)
Unknown	37 (22.6)
Stem cell source Peripheral blood	119 (72.6)
Bone marrow	9 (5.5)
Cord blood	3 (1.8)
Peripheral blood + bone marrow	3 (1.8)
Unknown	30 (18.3)
Donor type	
Related	106 (64.6)
Unrelated	38 (23.2)
Unknown	20 (12.2)
Tissue match	
Complete	99 (60.4)
Partial	21 (12.8)
Unknown	44 (26.8)
Biopsy-proven extra-GI GVHD	117 (71.3)
Skin	65 (39.6)
Liver	21 (12.8)
Oral mucosa	14 (8.5)
Eye	15 (9.1)
Lung	2 (1.2)
Gastrointestinal symptoms	
Nausea-vomiting	18 (11.0)
Diarrhea	50 (30.5)
Nausea-vomiting + diarrhea	40 (24.4)
Unknown	56 (34.1)
Endoscopic findings	
Normal mucosa	23 (33.8%)
Erythema	17 (25%)
Aphthous lesion	20 (29.4%)
Ulceration	8 (11.8%)
Time between transplantation and biopsy (days), median (range)	59 (10-1500)

**Table 3.** Summary of Previous Studies on GI GVHD

Author	Year	Case n	Frequency of GI GVHD	Severity of GI GVHD	Clinicopathologic Correlation
Ma et al^9^	2015	110	LGT > UGT Duo > stom Similar among LGT	LGT > UGT Similar in UGT LC > RC	–
Ross et al^8^	2008	112	LGT > UGT	–	–
Crowell et al^11^	2013	20	LGT > UGT	–	–
Thompson et al^12^	2006	24	LGT > UGT	–	No
Nydegger et al^10^	2007	11	Stom-Duo > Oes LGT > UGT	–	–
Aslanian et al^23^	2012	27	LGT = UGT Similar among LGT	–	–
Roy et al^24^	1991	77	LGT = UGT	–	–
Ip et al^13^	2016	46	Duo > Oes -Stom Similar among LGT	–	–
Nomura et al^25^	2017	186	Similar among LGT	T.ile > RC > LC	–
Minamino et al^26^	2015	16	Rec > T.ile	–	–
Abraham et al^5^	2014	210	–	–	No
Velasco-Guardado et al^31^	2012	197	–	–	Yes
Melson et al^32^	2007	23	–	–	Yes
Sauvestre et al^33^	2018	112	–	–	Low survival with higher pathologic grade
Present study	2023	164	LGT > UGT Duo > Oes-Stom Similar among LGT	LGT > UGT Duo > Oes-Stom Duo = LGT Similar among LGT	Low positive correlation

LGT, lower gastrointestinal tract; UGT, upper gastrointestinal tract; LC, left colon; RC, right colon; Duo, duodenum; Stom, stomach; Oes, esophagus; Rec, rectum; T.ile, terminal ileum.
